# Schizotypy and Behavioural Adjustment and the Role of Neuroticism

**DOI:** 10.1371/journal.pone.0030078

**Published:** 2012-02-20

**Authors:** Christoph Völter, Tilo Strobach, Désirée S. Aichert, Nicola Wöstmann, Anna Costa, Hans-Jürgen Möller, Torsten Schubert, Ulrich Ettinger

**Affiliations:** 1 Department of Psychology, University of Munich, Munich, Germany; 2 Department of Psychiatry, University of Munich, Munich, Germany; The University of Queensland, Australia

## Abstract

**Objective:**

In the present study the relationship between behavioural adjustment following cognitive conflict and schizotypy was investigated using a Stroop colour naming paradigm. Previous research has found deficits with behavioural adjustment in schizophrenia patients. Based on these findings, we hypothesized that individual differences in schizotypy, a personality trait reflecting the subclinical expression of the schizophrenia phenotype, would be associated with behavioural adjustment. Additionally, we investigated whether such a relationship would be explained by individual differences in neuroticism, a non-specific measure of negative trait emotionality known to be correlated with schizotypy.

**Methods:**

106 healthy volunteers (mean age: 25.1, 60% females) took part. Post-conflict adjustment was measured in a computer-based version of the Stroop paradigm. Schizotypy was assessed using the Schizotypal Personality Questionnaire (SPQ) and Neuroticism using the NEO-FFI.

**Results:**

We found a negative correlation between schizotypy and post-conflict adjustment (r = −.30, p<.01); this relationship remained significant when controlling for effects of neuroticism. Regression analysis revealed that particularly the subscale No Close Friends drove the effect.

**Conclusion:**

Previous findings of deficits in cognitive control in schizophrenia patients were extended to the subclinical personality expression of the schizophrenia phenotype and found to be specific to schizotypal traits over and above the effects of negative emotionality.

## Introduction

Patients with schizophrenia show impairments in conflict monitoring, error detection and behavioural adjustment following high conflict or errors [1]–[3]. The processes underlying such deficits have been termed *cognitive control*, e.g. [4].

Here, we investigated whether such deficits are also associated with the subclinical expression of the schizophrenia phenotype, i.e. schizotypy. For that purpose, we applied a modified version of the Stroop colour naming paradigm. In this paradigm, colour words (Red, Green, Blue) are presented in different ink colours. In congruent trials the colour word and the ink colour agree whereas in incongruent trials they do not. The basic finding - the Stroop effect – reflects the fact that subjects take longer to name the ink colour in incongruent trials than in congruent trials. This effect is thought to result from interference between word reading, which is considered as an automatic, overlearned process and colour naming, which is thought to be a volitional process. Interference presumably occurs by the competition between these two distinct processing pathways [4]. The Stroop task is therefore often considered to be an appropriate measure of cognitive control, particularly interference control [5]. In that respect, interference control refers to the inhibition of task-irrelevant distractors or internal stimuli that are interfering with the current task.

Interestingly, healthy subjects are known to increase the amount of cognitive control in difficult situations, for example in situations with interfering task characteristics or processing conflicts. Such increased cognitive control can be observed after the occurrence of high-conflict trials and is expressed as improved performance in the subsequent trial. In line with this, Kerns et al. [6] found - using the Stroop colour naming task - improved performance, i.e. shorter reaction times in incongruent trials following an incongruent trial (high conflict in previous trial) but not for incongruent trials following a congruent trial (low conflict in previous trial). Kerns [7] replicated these findings using the Simon task.

Importantly, Kerns and colleagues [3] found that schizophrenia patients do not show significant post-conflict and post-error trial-to-trial adjustments using the same paradigm. This behavioural abnormality was accompanied by reduced anterior cingulate cortex (ACC) activity, in line with previous functional magnetic resonance imaging (fMRI) studies indicating a major role of the ACC in the detection of conflict during information processing [6], [8]. Deficits in post-error adjustments in schizophrenia have also been found in other studies [1], [2].

Together, these studies indicate that schizophrenia patients have problems with conflict monitoring and post-conflict adjustments. An important question in this context is whether this finding can be extended to the subclinical manifestation of the schizophrenia phenotype. Studying schizotypy in healthy individuals has the advantage of allowing the investigation of schizophrenia spectrum traits whilst avoiding confounding factors like antipsychotic treatment or hospitalization often present in schizophrenia patients.

Schizotypy refers to state-independent, stable personality traits and is thought to be a multidimensional construct, e.g. [9]–[11]. The 3-factor model by Raine and colleagues [11], based on the schizotypal personality questionnaire (SPQ) [12], includes positive schizotypy, negative schizotypy, and cognitive disorganization; it was validated in several studies, e.g. [10], [13], [14]. Positive schizotypy refers to delusions and perceptual aberrations. Negative schizotypy is characterized by anhedonia, lack of volition and social anxiety. Finally, disorganized schizotypy is defined by disorganized speech and behaviour. Kerns [10] found that disorganized schizotypy in particular was related to deficits in measures of cognitive control.

Importantly, converging evidence indicates that schizotypy and schizophrenia are not only related on a phenomenological level but also on the neurocognitive level. Numerous studies have shown that schizotypic individuals have similar cognitive deficits as schizophrenia patients [15], [16]. However, it remains unclear whether post-conflict adjustments of cognitive control are associated with schizotypy. Therefore, the first aim of the present study was to answer this question.

An important issue in the schizotypy literature is the role of neuroticism. Neuroticism refers to negative emotionality or, as stated by Claridge and Davis [17], an “individual's emotional reactivity, tendency to worry, susceptibility to negative mood and proneness to psychopathology” (p. 384). There is considerable overlap between schizotypy and neuroticism in healthy subjects, e.g. [10], [18], [19], [20] and higher levels of neuroticism are found in schizophrenia [21]–[23] and schizotypal personality disorder (SPD) [24].

The exact causal relationship between schizotypy and neuroticism is still under debate: either neuroticism is considered as consequence of psychotic symptoms [25] or it is described as an unspecific predictor of various sorts of psychopathology, i.e. not restricted to psychoses [17]. Apart from that, it is crucial to investigate whether behavioral or cognitive deficits related to schizotypy are moderated by neuroticism or not [26]. A second aim of the present study, therefore, was to address this issue in relation to post-conflict adjustment.

We hypothesized based on previous findings from schizophrenia [3] that higher schizotypy would be associated with reduced post-conflict adjustment. In addition, we examined whether any such relationship would be explained by neuroticism. Finally, we investigated which of the schizotypy features are most strongly associated with deficits in post-conflict adjustment. On the basis of previous research on abnormal error detection and compensation in schizophrenia [27] we hypothesized that negative features would be most strongly associated with such deficits.

## Methods

### Participants

One hundred and six volunteers were recruited through local advertisements and circular emails. Participants were screened for clinical exclusion criteria and provided demographic and psychometric personality information. Clinical exclusion criteria were (1) any current psychiatric diagnosis (using the German version of the Mini-International Neuropsychiatric Interview [28]), (2) history of neurological complications, (3) history of psychosis or ADHD in first-degree relatives, (4) any current physical condition, (5) any current consumption of over-the-counter medication, prescription medication (except for contraceptives) or psychotropic drugs, (6) colour vision deficiency. In addition, subjects whose first language was not German were excluded. All subjects completed the study and met the criteria. The study was approved by the ethics committee of the Faculty of Medicine of the University of Munich (Ethikkommission der Medizinischen Fakultät der LMU). Subjects provided written informed consent and were reimbursed for their participation.

### Psychometric Assessment

Demographic data were collected using a questionnaire asking among other for age, gender, and years of education.

Schizotypy was assessed using the German version of the schizotypal personality questionnaire (SPQ) [12], [29]. The SPQ is a self-report scale based on DSM-III-R criteria for SPD [30]. It contains 74 items with a two-point response format (yes/no) yielding subscales for the nine different DSM-III-R schizotypal traits: Ideas of Reference (IR, 9 items), Social Anxiety (SA, 8 items), Magical Thinking (MT, 7 items), Unusual Perceptual Experiences (UPE, 9 items), Eccentric Behaviour (EB, 7 items), No Close Friends (NCF, 9 items), Odd Speech (OS, 9 items), Constricted Affect (CA, 8 items) and suspiciousness (S, 8 items). Thereby, IR, MT, UPE and S refer to positive schizotypy whereas SA, NCF and CA refer to negative schizotypy. Finally, disorganization is defined by the SPQ subscales EB and OS. The SPQ questionnaire has high internal reliability (.91), test-retest reliability (.82) and validity [12]. In the present sample the SPQ had high internal reliability (.91). The items were coded as 1 for “yes” and 0 for “no”. For the total SPQ mean score, the mean value across all 74 items was calculated; for the 9 subscales the mean score was calculated across the corresponding items.

Neuroticism (N) was assessed using the German version of the NEO-FFI [31], consisting of 12 items. The items use a five-point Likert-type response format (“strongly disagree” = 0; “strongly agree” = 4). In the current sample the N subscale had high internal reliability (.82).

Verbal IQ was estimated using the MWT-B [32]. In this test subjects are required to identify a real word among four non-words in each row. A correct answer was coded as 1, an incorrect answer as 0. A maximal score of 37 could be obtained.

### Procedure

The stimuli consisted of the German words “ROT” (red), “GRÜN” (green) or “BLAU” (blue) shown in either red, green or blue ink, on a notebook computer (Dell Latitude, D430) with a 12.1-inch screen. Trials were considered either congruent or incongruent depending on whether word and ink colour were the same (e.g., the word “ROT” in red ink) or not (e.g., the word “ROT” in green ink). Subjects were instructed to name the ink colour as fast and accurately as possible by pressing the arrow keys “left”, “down”, “right” with their right ring finger, middle finger, and index finger, respectively. In the beginning of every trial there was a fixation cross (approximately 0.6° of visual angle; distance to screen approx. 57 cm) presented in the centre of the screen for 1 sec followed by the colour word (vertical size approx. 0.8°, horizontal size approx. 3.0° of visual angle) that stayed on the screen for a maximum of 2 sec or until the subject pressed a button. After a pause of .5 sec, a new trial began. There were two blocks of 99 trials each with a short break between blocks. In total, there were 138 congruent trials (69.7%) and 60 incongruent trials (30.3%). In the beginning of each block 6 congruent trials were presented in succession to increase the interference effect (see Kerns et al. [6]). These 12 trials across both blocks were excluded from further analysis. All other trials were presented in a randomized order. The experiment took about 12 minutes.

### Statistical Analysis

Statistical analysis was carried out using the Statistical Package for the Social Sciences, Release 17.0 (SPSS Inc., Chicago, IL). For the initial analysis of post-conflict adjustment the significance level was set at p<.05 and p-values between .05 and .1 were considered trends. For the subsequent correlation analyses of the SPQ scale and subscales the significance level was set at 0.005 to account for multiple comparisons using the Bonferroni correction; p-values between .005 and .01 were considered trends. Following Kerns et al. [6], only trials that did not represent either an ink colour or word repetition were used. This procedure controls for partial priming effects between subsequent trials, which may be related to repetition of word or colour information between trials [33]. Additionally, error and post-error trials were excluded to account for effects of post-error slowing (on average 7 trials were excluded per subject). Trials were categorized according to congruency and incongruency in the current and in the previous trial. Consequently, there were two factors yielding trials from four categories: congruent trials preceded by congruent trials (cC), congruent trials preceded by incongruent trials (iC), incongruent trials preceded by congruent trials (cI) and incongruent trials preceded by incongruent trials (iI). Reaction time (RT) data were analyzed by repeated measures analysis of variance (ANOVA) with the factors “congruency of present trial” and “congruency of previous trial”. On the basis of significant interactions, post-hoc t-tests using Bonferroni-correction were calculated.

The basic Stroop effect was calculated with the following formula: RT (incongruent current trials)−RT (congruent current trial). Post-conflict trial-to-trial adjustment was calculated identical to Kerns et al. [3]: [RT(iC)−RT(cC)]+[RT(cI)−RT(iI)], i.e. the higher this adjustment score, the more the participants improved their performance in the post-conflict trial.

Pearson correlations were carried out to assess associations between post-conflict adjustment and schizotypy and between schizotypy and N. Additionally, in order to control for possible effects of age, sex, years of education, verbal IQ, and N partial correlations were carried out. Thereafter, a linear regression model was calculated to predict post-conflict adjustment from the 9 SPQ subscales. The subscales were introduced as predictors in one block using the stepwise method (probability for entry set at .05) in order to identify the strongest predictors of post-conflict adjustment.

In a further regression analysis, we investigated the impact of N on the relationship between SPQ subscales and post-conflict adjustment. We first introduced Neuroticism as covariate in the regression model. In a second step we introduced the subscales using the stepwise method (entry probability set at .05).

SPQ mean score was positively skewed (skewness = 1.63). A square root transformation normalized the skew (0.31). Using square root transformed scores instead of untransformed variables did not change the pattern of significant findings with regards to post-conflict adjustment. For that reason, and to make the correlation results more compatible with the descriptive data, untransformed variables were used in all analyses presented here.

## Results

Data of six subjects were excluded from the analysis: three subjects due to poor behavioural performance (error rate >50%), and three subjects due to extreme post-conflict adjustment values that deviated more than 3 SD from the mean. Demographic and behavioural data are presented in Table 1.

**Table 1:** Demographic and behavioural data (± SEM).

N	100
Age (Years)	25.1±0.5
Female, %	60.0
Years of Education	16.2±0.2
Mean NEO-FFI Neuroticism Subscore	1.4±0.1
Total MWT-B Score	30.9±0.3
Post-Conflict Adjustment, msec	21.4±9.6
Stroop Effect, msec	152.7±11.2
Error Rate in Congruent Trials, %	2.4±0.3
Error Rate in Incongruent Trials, %	9.4±0.9

NEO-FFI Neuroticism subscore refers to the mean value with a minimum of 0 and a maximum of 4. Total MWT-B Score has a maximum of 37.

### Behavioural adjustments following high conflict

**Figure 1:**
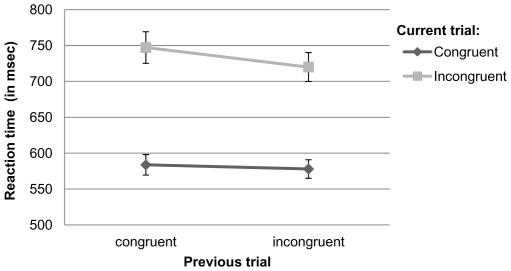
Post-conflict trial-to-trial adjustment. Data represent reaction times (RT) in msec (±SEM). Congruent and incongruent current trials are shown as a function of previous trial congruency. RTs in current incongruent trials were significantly shorter when preceded by an incongruent trial than by a congruent trial.

A 2×2 (congruency of current trial×congruency of previous trial) repeated measures ANOVA showed a significant main effect of current trial congruency on reaction times (F[1,99] = 187.21; p<.001; partial η^2^ = .65), reflecting the Stroop effect with longer reaction times in incongruent than congruent trials. In addition, there was a significant main effect of previous trial congruency (F[1,99] = 6.32; p<.05; partial η^2^ = .06), reflecting shorter reaction times in trials preceded by an incongruent trial. Crucially, we found a significant interaction between previous trial congruency and current trial congruency (F[1,99] = 4.93; p<.05; partial η^2^ = .05). Bonferroni corrected t-tests showed that there was no effect of previous trial on the reaction times in current congruent trials (p>.1); however, there was a significant influence of previous trial type on reaction times in current incongruent trials (p<.01) reflecting the occurrence of post-conflict adjustment (Figure 1).

### Relationship between SPQ and Post-Conflict Adjustment

We found a significant negative correlation between mean SPQ score and post-conflict adjustment (r = −.30; p<.005) indicating greater post-conflict adjustment in lower schizotypy. Importantly, the correlation remained marginally significant after introducing sex, age, years of education, neuroticism (N) and MWT-B verbal IQ as covariates (r = −.29; p = .005). In contrast, there was no significant correlation between N and post-conflict adjustment (r = −.07; p = .50).

Pearson correlations of schizotypy subscales (for mean scores see Table 2) with post-conflict adjustment and N are presented in Table 3. No Close Friends (NCF) was significantly correlated with post-conflict adjustment (p<.005); Eccentric Behavior (EB) was associated on trend level (p<.01). When controlling for N the correlation between post-conflict adjustment and NCF (r = −.29; p<.005) remained significant; the association of EB (r = −.27; p<.01) still reached trend level. The only SPQ subscale that was significantly correlated with N was Social Anxiety (r = .45; p<0.001).

**Table 2:** Mean values (± SEM) of SPQ total score and subscales.

SPQ Scales	Score	Cronbach's alpha
Mean SPQ Score	0.12±0.01	0.91
Ideas of Reference	0.15±0.02	0.75
Social Anxiety	0.13±0.02	0.69
Magical Thinking	0.05±0.01	0.75
Unusual Perceptual Experiences	0.05±0.01	0.53
Eccentric Behaviour	0.12±0.02	0.82
No Close Friends	0.09±0.01	0.66
Odd Speech	0.20±0.02	0.78
Constricted Affect	0.11±0.02	0.58
Suspiciousness	0.14±0.01	0.44

Scores refer to the mean values that have a minimum of 0 and a maximum of 1.

**Table 3:** Pearson correlations between post-conflict behavioural adjustments and NEO-FFI Neuroticism and SPQ subscales.

	Post-Conflict Adjustment		NEO-FFI Neuroticism	
SPQ Scales	r	p(2-tailed)	r	p(2-tailed)
Mean SPQ Score	−0.30	0.003*	0.24	0.02
Ideas of Reference	−0.21	0.04	0.13	0.20
Social Anxiety	−0.15	0.13	0.45	<0.001*
Magical Thinking	−0.17	0.10	−0.03	0.79
Unusual Perceptual Experiences	−0.20	0.05	0.21	0.04
Eccentric Behaviour	−0.27	0.008(*^)^	−0.05	0.60
No Close Friends	−0.30	0.003*	0.23	0.02
Odd Speech	−0.15	0.13	0.13	0.18
Constricted Affect	−0.19	0.06	0.18	0.08
Suspiciousness	−0.13	0.19	0.22	0.03

( *^)^p<.01 and *p<.005 indicate levels of statistical significance following Bonferroni correction.

Additionally, we conducted a regression analysis to assess which of the SPQ subscales best predicted post-conflict adjustment. NCF was the only predictor (R = .30). For the final model adjusted R^2^ was .08 (F[1,98] = 9.37, p<.005). Low post-conflict adjustments were associated with high NCF scores (*Beta* = −.30). The introduction of N in the first step did not affect these findings, i.e. NCF still predicted post-conflict adjustment (Table 4).

**Table 4:** Summary of linear regression analysis of post-conflict adjustment.

	Predictors	Beta	r^2^	F	p	df
Model 1	NCF	−0.30	0.08	9.37	0.003	1/98
Model 2: 1st Step	N	−0.07	0.01	0.47	0.50	1/98
Model 2: 2nd Step	NNCF	0.00−0.30	0.07	4.64	0.012	2/97

Model 1: SPQ subscales introduced using stepwise method (entry probability: .05); Model 2: N entered in first step, SPQ subscales introduced in second step using stepwise method; NCF: No Close Friends SPQ subscale; N: NEO-FFI neuroticism.

## Discussion

In the present study we investigated the relationship between behavioural adjustment in cognitive control and schizotypy. A secondary aim was to investigate whether any such observation could be accounted for by individual differences in negative trait emotionality, or neuroticism.

We first replicated the experimental effect observed by Kerns et al. [6]: post-conflict behavioural adjustment (in incongruent trials following an incongruent trial) was found in a Stroop task while excluding potential effects of stimulus priming. Post-conflict adjustment has been explained by the conflict monitoring hypothesis [4] according to which response conflict elicits enhanced top-down control which leads to performance improvements on subsequent trials.

In line with our individual differences hypothesis we found that there was an association of higher levels of schizotypy with reduced levels of post-conflict trial-to-trial adjustment. Our result extends previous findings showing deficits in post-conflict adjustment in schizophrenia patients [3]. Thus, the impairment in schizophrenia described by Kerns et al. [3] is likely not a mere consequence of the illness manifestation as an association is observed here in a subclinical spectrum population that does not suffer from potential confounds like medication, long-term hospitalisation and illness artefacts. Such observations of neural or cognitive impairments in schizotypy resembling those seen in schizophrenia provide support for the assumption of a continuum of this personality trait to the clinical disorder [9].

Importantly, the correlation between schizotypy and behavioural adjustment could not be accounted for by individual differences in neuroticism. Neuroticism is a global measure of negative emotionality and includes items on depressiveness and anxiety. Neuroticism has been found to be elevated in a number of personality disorders and psychiatric conditions, e.g. [21], [24], [34], [35]. Specifically, a number of studies have observed sizeable correlations between neuroticism and schizotypy, e.g. [10], [19], [20] making it important to examine the specificity of associations observed between schizotypy and measures of cognitive control (see also [18], [36]). The present results indicate that the association of schizotypy with cognitive control is observed over and above any effects of neuroticism, suggesting specificity of such cognitive deficits to schizophrenia spectrum traits.

While the neural mechanisms underlying the observed association between behavioural adjustment and schizotypy remain unclear, Kerns and colleagues [3] found that anterior cingulate cortex (ACC) activity predicting behavioral adjustment was reduced in schizophrenia patients. From the present results we would expect that in highly schizotypal individuals the conflict-related ACC activity would similarly be reduced compared to less schizotypal individuals. Evidence for the existence of such brain functional similarity between schizotypy and schizophrenia comes from a recent fMRI study of the antisaccade task showing that higher levels of schizotypy are associated with reductions in activity in brain regions also found to be of reduced activity in schizophrenia [37]. Interestingly, Hazlett and colleagues [38] recently reported reduced ACC grey matter volume in both schizophrenia and schizotypal personality disorder, strengthening the hypothesis of ACC mediated dysfunction in behavioural adjustment in schizotypy. Future studies might aim to investigate whether functional schizotypy-related abnormalities occur in the ACC during performance monitoring.

An important methodological consideration concerning this study is that the mean SPQ score in the current community-based sample was comparatively low (0.12±0.11). However, it should be noted that the mean SPQ scores of the German version of the SPQ scale generally tend to be lower (0.29±0.14; Klein et al. [29]) than in the original English version (0.36±0.15; Raine et al. [11]). Additionally, in contrast to the studies by Raine et al. and Klein et al., strict exclusion criteria such as any current psychiatric diagnosis in the Mini-International Neuropsychiatric Interview [39] were used for the present study, possibly leading to the exclusion of high schizotypals. In line with that assumption, another study that also used the Mini-International Neuropsychiatric Interview for screening of participants reported similarly low SPQ scores 0.15±0.11 [40]. Thus, while our study only included participants from the low-to-medium spectrum of schizotypy the results may be interpreted to support the validity of the fully dimensional approach [41]. An advantage of this approach is that confounding factors such as drug abuse in extreme scorers can be avoided.

Fine grained correlation analyses of SPQ subscales revealed that the subscales No Close Friends (NCF) and Eccentric Behaviour (EB), features of negative and disorganized schizotypy, respectively, were associated with post-conflict adjustment. In a subsequent regression analysis, No Close Friends was the only predictor of post-conflict adjustment, underscoring the role of negative schizotypy in cognitive control.

In contrast to the present study, previous research on schizotypy instead found disorganized factors to impact on cognitive control [10]. However, it should be noted that the method of assessing cognitive control differs between studies: whereas Kerns [10] measured cognitive control using measurements of prepotent inhibition (Stroop, Simon, and Preparation for Overcoming a Prepotent Response task) the present study focused on trial-to-trial post-conflict adjustments. Such behavioural adjustments involve not only response inhibition but also monitoring of response conflict and engagement of compensation processes [6]. By recruiting these cognitive control processes subjects improve their performance, i.e. overcome response interference in a subsequent trial. Thus, the present correlation between post-conflict adjustment and schizotypy might be due to either conflict monitoring, engagement of control processes or both. Importantly, Bates and colleagues [27] have shown using a go/no-go task that error-related negativity, an electromyographic potential associated with error detection and compensation processes leading to adjustments in behaviour [42], are correlated with negative schizophrenia symptoms. In contrast, correct response negativity, an electromyographic correlate of performance monitoring (not involving error detection) [43], was correlated with disorganized schizophrenia symptoms. In this context it is also important to note that previous research has shown that error- and conflict-related activity is decreased in schizophrenia patients in the same area of the ACC suggesting similar underlying neural mechanisms [3]. The current results are in line with these findings: conflict detection and adjustment processes in cognitive control were correlated primarily with a negative feature of schizotypy, namely, No Close Friends.

One question that arises is why the No Close Friends subscale in particular predicted behavioural adjustment. Across different samples and studies it has been shown that this subscale exhibited the highest loadings on negative schizotypy ranging from .73 to .89 [11], [13]. Therefore, No Close Friends can be considered as a core feature of negative schizotypy. High scores on No Close Friends items (e.g., I find it hard to be emotionally close to other people) are indicative of interpersonal deficits. Interestingly, Holmes and Pizzagalli [44] recently found - using the same post-conflict adjustment assessment as in the present study - impaired post-error and post-conflict adjustments in participants with elevated depressive symptoms on the Beck Depression Inventory (BDI). This impairment was especially pronounced when emotionally negative feedback was given. From the present findings one might argue that interpersonal deficits that also play a role in depression underlie this association. In line with this notion, the Social Anhedonia Scale (which also measures interpersonal deficits and even shares an item with NCF) and BDI score have been found to be significantly correlated (r = .36) [45].

In summary, the present investigation shows that schizotypy is associated with post-conflict behavioural adjustment. This finding mirrors and extends the observation that patients with full-blown schizophrenia show reduced post-conflict adjustment on this measure. Our data suggest that deficits in behavioural adjustment are present in a subclinical spectrum population of schizophrenia, making this measure a promising schizophrenia spectrum marker.
